# Identifying ATP-Binding Cassette Member B5 as a New Biomarker for Oral Squamous Cell Carcinoma

**DOI:** 10.32604/or.2025.064276

**Published:** 2025-07-18

**Authors:** Li Yu, Xiaoyan Zhang, Yan Feng, Xinyue Liao, Tiejun Zhou, Hang Si, Yun Feng, Decai Wang, Yongxian Lai

**Affiliations:** 1 Oral & Maxillofacial Reconstruction, Regeneration of Luzhou Key Laboratory, Luzhou, 646000, China; 2 Department of Periodontics & Oral Mucosal Diseases, The Affiliated Stomatology Hospital of Southwest Medical University, Luzhou, 646000, China; 3 NHC Key Laboratory of Nuclear Technology Medical Transformation, Mianyang Central Hospital, Mianyang, 621000, China; 4 Department of Pediatric Dentistry, The Affiliated Stomatology Hospital of Southwest Medical University, Luzhou, 646000, China; 5 Department of Pathology, The Affiliated Hospital of Southwest Medical University, Luzhou, 646000, China; 6 Department of Urology, Mianyang Central Hospital, School of Medicine, University of Electronic Science and Technology of China, Mianyang, 621000, China; 7 Department of Preventive Health Care, The Affiliated Stomatology Hospital of Southwest Medical University, Luzhou, 646000, China

**Keywords:** Oral squamous cell carcinoma (OSCC), ATP-binding cassette subfamily B member 5 (ABCB5), migration, invasion, epithelial-mesenchymal transition (EMT)

## Abstract

**Background:**

Oral squamous cell carcinoma (OSCC) is the most common head and neck malignancy with a low five-year survival rate. ATP-binding cassette subfamily B member 5 (ABCB5) has been linked to tumorigenesis. However, its role in inducing OSCC remains unclear.

**Methods:**

Quantitative reverse transcription polymerase chain reaction (qRT-PCR), western blot, and immunocytochemistry (ICC) were performed to examine the level of ABCB5 in OSCC (CAL27 and HSC-3) and human oral keratinocyte (HOK). ABCB5 was knocked down in CAL27 cells using ABCB5-specific small interfering RNA (ABCB5 siRNA), and its contribution to migration, invasion, and epithelial-mesenchymal transition (EMT), a process by which epithelial cells lose their tight junction and acquire an increased migratory and invasive phenotype resembling that of mesenchymal cells, were evaluated by three-dimension and transwell migration and invasion assays, qRT-PCR and ICC. An *in vivo* OSCC model was established using 4-nitroquinoline-1-oxide (4NQO), a carcinogenic chemical that is commonly used to develop OSCC by destroying DNA synthesis and oxidative stress. Pathological alterations, ABCB5, and EMT markers were evaluated by H&E staining, immunohistochemistry, and qRT-PCR.

**Results:**

ABCB5 was significantly upregulated in CAL27 and HSC-3 cells as compared to HOK. Knockdown of ABCB5 significantly reduced the number of migrated and invaded CAL27 cells, accompanied by the significantly increased E-cadherin and decreased Vimentin and N-cadherin under Transforming growth factor β (TGF-β) treatment. *In vivo*, as OSCC advanced, a notable rise in the expressions of ABCB5, N-cadherin, and Vimentin, while a statistical decrease in E-cadherin was demonstrated.

**Conclusion:**

ABCB5 promotes the migration, invasion, and EMT of OSCC. ABCB5 might be a new biomarker and potential therapeutic target for OSCC.

## 1 Introduction

Oral squamous cell carcinoma (OSCC), the predominant subtype of head and neck squamous cell carcinoma [1,2], predominantly affects individuals between 55 and 64 years old [3]. The most common sites that embed the OSCC are the buccal, tongue, palate, and floor of the mouth mucosa. The incidence of OSCC is high, with over 630,000 new cases diagnosed annually worldwide [4]. However, due to the invasiveness and metastasis, the overall five-year survival rate of OSCC remains less than 50% [5,6]. Several biomarkers, including tumor protein 53 (TP53) [7], epidermal growth factor receptor (EGFR) [8], Cyclin D1 [9], Ki67 [10], prognostic biomarkers such as hypoxia-inducible factor (HIF)-1α [11], programmed death-ligand 1 (PD-L1) [12], and epithelial-mesenchymal transition (EMT) markers [13] have been identified for the diagnosis and prognosis of OSCC [14–16].

ABC transporters are a superfamily of transmembrane proteins, which were divided into subgroups from A to G. ABC transporters play a fundamental role in the progression of OSCC by modulating drug efflux [17–19], tumor metastasis [20], and cancer stem cell maintenance [21]. Overexpression of ATP-binding cassette subfamily B member 1 (ABCB1) [22], ATP*-*binding cassette subfamily C member 1 (ABCC1) [23], and ATP-binding cassette subfamily G member 2 (ABCG2) [24] in OSCC increased the resistance to cisplatin, paclitaxel, and other chemotherapeutic agents. In cancer stem cells (CSCs), ABC transporter extruded out the toxic and xenobiotic substances thereby mediating chemoresistance [18]. ATP-binding cassette subfamily C member 2 (ABCC2) was associated with chemoresistance of tumors [25]. In addition, high expression of ABCB1 indicated a poor prognosis of cancer [26]. ATP-binding cassette subfamily B member 5 (ABCB5) is a family member of ABC transporter, located on chromosome 7p15 and consists of 16 exons (108 kb) with 812 amino acids [17]. CSCs mediate the tumor growth, invasiveness, chemoresistance, and recurrence of multiple types of malignancies. CSCs of non-small cell lung cancer (NSCLC), Merkel cell carcinoma, and colorectal cancer (CRC) showed high expression of ABCB5 [27,28]. Research has shown that ABCB5 regulates the EMT and invasion of CRC by regulating receptor tyrosine kinases (RTKs, AXL) [29]. In breast cancer, elevated ABCB5 expression was connected to enhanced migration and EMT by upregulating Zinc finger E-box binding homeobox 1 [30]. Furthermore, high expression of ABCB5 was responsible for chemoresistance in melanoma cells [31].

EMT denotes a process wherein epithelial cells become capable of migrating as mesenchymal cells and lose their capacity to adhere [5]. EMT is the hallmark of cancer [32,33]. Wnt/β-catenin signaling pathway promoted EMT of NSCLC [33], whereas the phosphatidylinositol 3-kinase/serine/threonine protein kinase B/mechanistic target of rapamycin (PI3K/Akt/mTOR) signaling pathway was involved in initiating EMT in OSCC [32]. Furthermore, it has been documented that the extracellular regulated protein kinase (ERK) and c-Jun N-terminal kinase (JNK) pathways control EMT progression in CRC [34]. A study revealed that EMT functions in facilitating the proliferation, migration, and invasion of OSCC cells [35]. In normal and premalignant epithelial cells, the tumor growth can be inhibited by Transforming growth factor β (TGF-β), a multifunctional cytokine [36]. While in cancer cells, TGF-β functions as a key regulator to induce EMT by regulating the expression and activity of ERK, Smad and non-Smad signaling pathways [37,38].

Compared to normal and inflammatory hyperplasia tissues, the level of ABCB5 in OSCC was significantly elevated [17,39]. However, the mechanistic role of ABCB5 in regulating OSCC progression has yet to be determined. This study investigated the functional role of ABCB5 in regulating the migration and invasion of OSCC. Moreover, we explored the role of ABCB5 in promoting OSCC using a 4-nitroquinoline-1-oxide (4NQO) carcinogen mouse model *in vivo*.

## 2 Materials and Methods

### 2.1 Cell Culture Conditions and Reagents

The human OSCC cell lines HSC-3 (Otwo Biotech, HTX2055, Shenzhen, China) and CAL27 (Shanghai Yaji Biotechnology Co., Ltd., BFN60700437, Shanghai, China), and Human Oral Keratinocyte (HOK) (Shanghai Jingfeng Biotechnology Co., Ltd., GT2367C, Shanghai, China) were cultured in Dulbecco’s Modified Eagle’s Medium (DMEM) (Procell Life Science & Technology Co., Ltd., PM150210, Wuhan, China) with 10% fetal bovine serum (FBS) (Beijing Secret Jiade Medical Technology Co., Ltd., 11011-8611, Beijing, China) and 1% Gibco™ penicillin & streptomycin (Thermo Fisher Scientific, 15070063, Shanghai, China) at 37°C in a 5% CO_2_ incubator. The absence of Mycoplasma pulmonis was confirmed by Polymerase Chain Reaction (PCR) with Venor^®^ Gem Classic (DIYI BIO, 11-1025G, Beijing, China).

### 2.2 Quantitative Reverse Transcription Polymerase Chain Reaction (qRT-PCR)

The total ribonucleic acid (RNA) from tissues or cells was obtained using TRIzol (Thermo Fisher Scientific, 15596018CN). TaqMan™ Reverse Transcription Reagents (Thermo Fisher Scientific, 4304134) were utilized for synthesizing complementary deoxyribonucleic acid (cDNA) with the conditions of 37°C for 15 min, and 85°C for 5 s. DyNAmo HS SYBR Green qPCR Kit (Thermo Fisher Scientific, F410L) was used for cDNA synthesis. The primers utilized were listed in Table S1. The qRT-PCR procedure consisted of 40 cycles of predenaturation at 95°C for 2 min, denaturation at 95°C for 30 s, annealing at 60°C for 30 s, extension at 65°C for 2 min, and an additional extension period of 5 min at 70°C after the last cycle. The qRT-PCR results were normalized to Glyceraldehyde-3-phosphate dehydrogenase (GAPDH) or Gapdh. 2^−ΔΔCt^ was used to for quantitative calculation.

### 2.3 Western Blot

The CAL27, HSC-3, and HOK cells were collected after phosphate-buffered saline (PBS) wash. Proteins were isolated from the cells using radioimmunoprecipitation assay (RIPA) buffer (high) (Solarbio, R0010, Beijing, China) and extraction buffer supplemented with 1% sodium dodecyl sulfate (SDS) (Solarbio, S8010) and 1× halt protease and phosphatase inhibitor cocktail (Beijing Pulilai gene Technology Co., Ltd., P1261, Beijing, China). Then, the bicinchoninic acid (BCA) protein assay kit (Thermo Fisher Scientific, 23225) was employed to measure protein concentration. After being separated by sodium dodecyl sulfate-polyacrylamide gel electrophoresis (SDS–PAGE, Sangon Biotech, C671100-0060, Shanghai, China), the proteins were transferred to nitrocellulose membranes (Thermo Fisher Scientific, IB23001). After blocking with 5% skimmed milk, the primary ABCB5 antibody (Santa Cruz Biotechnology, Inc., sc-515910, Shanghai, China) was incubated with a dilution of 1:500 for an entire night at 4°C. Then, the Goat anti-Rabbit IgG (H + L) HRP secondary antibody (Oriscience Biotechnology Co., Ltd., PD302, Sichuan, China) at a dilution of 1:300 was incubated for 1 h at room temperature. β-actin (Jiangsu Kinke Biological Research Center Co., Ltd., AF7018, Jiangsu, China) served as a loading control. The ECL kit (Solarbio, PE0010) was used to image the protein bands. The ImageJ software (NIH, Bethesda, MD, USA) was used to perform the density analysis of the above protein bands.

### 2.4 Immunocytochemistry (ICC)

Immunocytochemical staining was conducted to analyze ABCB5 protein levels in CAL27, HSC-3, and HOK cells. In addition, E-cadherin and Vimentin protein levels were assessed in CAL27 cells after siABCB5 (Genepharma, Shanghai, China) transfection with or without TGF-β (ACROBiosystems, GMP-TG1H25, Beijing, China) treatment. After the indicated treatment, cells were treated with 4% paraformaldehyde (PFA) (Sangon Biotech, E672002-0001) and subsequently permeabilizated with 0.1% TritonX-100 (Sigma-Aldrich, T8787, Zhejiang, China) for 15 min, then, 5% bovine serum albumin (BSA) blocking buffer (Solarbio, SW3015) was used to block the antigen for 40 min at 37°C. Then, cells were incubated with the primary antibodies of ABCB5 (Thermo Fisher Scientific, PA5-72966, diluted 1:100), E-cadherin (Wuhan Fenn Biotechnology Co., Ltd., FNab02618, Wuhan, China, diluted 1:50), and Vimentin (Invitrogen, PA1-16759, Shanghai, China, diluted 1:300) overnight at 4°C. Followed by incubation with Goat anti-Rabbit IgG (H + L) Alexa Fluor 594 (Oriscience, PD306, Sichuan, China) or Goat anti-Rabbit IgG (H + L) Alexa Fluor 488 (Oriscience, s0008, Sichuan, China) antibodies 1 h at room temperature and counterstained with 4^′^,6-diamidino-2-phenylindole (DAPI) (Solarbio, PD304, Beijing, China). The images were photographed under the BX-53 orthogonal fluorescence microscope (Olympus Corporation, Japan) and quantified using the ImageJ software (NIH). 5 fields per group.

### 2.5 Transfection of siRNA

The CAL27 cell, which presented higher ABCB5 expression, was transfected with control small interfering RNA (siRNA) (5^′^-UUCUCCGAACGUGUCACGUTT-3^′^) or ABCB5 siRNA #1 (5^′^-GCAUGGAGGAUGCUUUGAATT-3^′^) (GenePharma) and siRNA #2 (5^′^-CAGGGAUACCUCUCCUCAATT-3^′^) (GenePharma) with Lipofectamine 3000 (Invitrogen, L3000015) in a ratio of 1:2. The transfected cells were cultured at 37°C with 5% CO_2_ for 6 h, then the transfection medium was removed, and the transfected cells were cultured in 15% FBS (without penicillin-streptomycin solution) culture medium at 37°C with 5% CO_2_ for 48 h. The knockdown effect of ABCB5 was achieved through qRT-PCR and ICC. The transfected cells were collected for subsequent experiments.

### 2.6 Two-Dimensional (2D) or Three-Dimensional (3D) Spheroid Migration and Invasion Assays

In the 2D transwell migration assay, cells supplied in serum-free medium were loaded into the upper chamber, while 10% FBS was added to the lower chamber. The under-membrane-migrated cells were fixed and stained with hematoxylin (Solarbio, G1150). For invasion evaluation, Matrigel (YEASEN, 40183ES08, Shanghai, China) was used to precoat the upper chamber membrane prior to the 2D transwell invasion assay. The procedure was then in accordance with the transwell migration assay. The number of migrated or invaded cells was calculated via ImageJ software (NIH). For the 3D spheroid migration and invasion assays, the cells were initially cultured in U-bottom non-adhesive plate and allowed to incubate overnight. Then, the 3D spheroid was transferred to a flat bottom plate for migration with an FBS-free culture medium. For the invasion assay, the flat bottom plate was precoated with Matrigel prior to the invasion assay. Images were performed at 0, 12, 24, 48, and 72 h to observe the number of cells climbing out of the 3D spheroid and quantified via ImageJ (NIH).

### 2.7 TGF-β Treatment

10^5^ cells CAL27 and HSC-3 cells were cultured for 24 h and stimulated with human recombinant TGF-β (ACROBiosystems, GMP-TG1H25, Beijing, China) at the concentrations of 0, 1, 2, and 5 ng/mL for 24 h. Then, cells were collected for the subsequent experiments.

### 2.8 Establishment of the 4NQO Carcinogen Mouse Model and Histological Analysis

The 4NQO is a carcinogenic chemical that is commonly used to develop OSCC by destroying DNA synthesis and oxidative stress. Therefore, to study the role of ABCB5 in forming OSCC, 4NQO was used to establish an *in vivo* OSCC model [40]. Six-week-old female 20 g C57BL/6 mice (Sichuan Yaokang Biotechnology Co., Ltd., cyhc0845, Sichuan, China) were randomly allocated into 4NQO group (*n* = 20) and control group (*n* = 6). As previously described [41], the mice in the 4NQO group were given drinking water supplemented with 50 µg/mL 4NQO (Macklin, N814871, Shanghai, China) to induce OSCC, while the mice in the control group were given normal drinking water. The mice were housed under specific-pathogen-free conditions (19°C–23°C, 30%–70% humidity) with a regular 12-h light/dark cycle. At 16, 18, 20, 22, and 24 weeks, 4 mice were randomly euthanized, and tongue tissues were collected and embedded in paraffin or optimal cutting temperature (OCT) compound (Solarbio, G6059) for further analysis. Hematoxylin–eosin (H&E) staining was performed for pathological analysis, and the samples were grouped by 2 experienced pathologists according to pathological findings. The ARRIVE Guidelines 2.0 for reporting animal research have been strictly followed in this study.

### 2.9 Immunohistochemistry (IHC)

Paraffin-embedded mouse tongue specimens were dewaxed and rehydrated, followed by antigen retrieval. The endogenous peroxidase in tissues was inhibited with a 3% H_2_O_2_ solution (Serve Life Science, MM0750-500ML, Shanghai, China) at room temperature for 10 min. Then, tongue tissues were blocked with 5% goat serum (Fuzhou Feijing Biotechnology Co., Ltd., PH0424, Fujian, China) and incubated with ABCB5 antibody (Thermo Fisher Scientific, PA5-72966, diluted 1:200) overnight at 4°C. Goat anti-rabbit IgG (H + L) HRP secondary antibody (Oriscience Biotechnology Co., Ltd., PD302, Sichuan, China, diluted 1:300) was used the next day at 37°C for 1 h and counterstained with hematoxylin. Tissue images were visualized using the BX-43 upright optical microscope (Olympus Corporation). ABCB5-positive regions were quantitatively analyzed using ImageJ software (NIH). 5 fields per group.

### 2.10 Statistical Analysis

All quantitative data were presented as the means ± standard deviations (SD). Statistical significance between two groups was determined by Student’s *t-*test, and one-way analysis of variance (ANOVA) or two-way ANOVA were used for multiple group comparisons. Statistical analysis was performed via Statistical Product and Service Solutions (SPSS) 27 software (IBM, New York, USA). *p* < 0.05 was considered statistically significant.

## 3 Results

### 3.1 ABCB5 Is Highly Expressed in Oral Cancer Cell Lines

**Figure 1:**
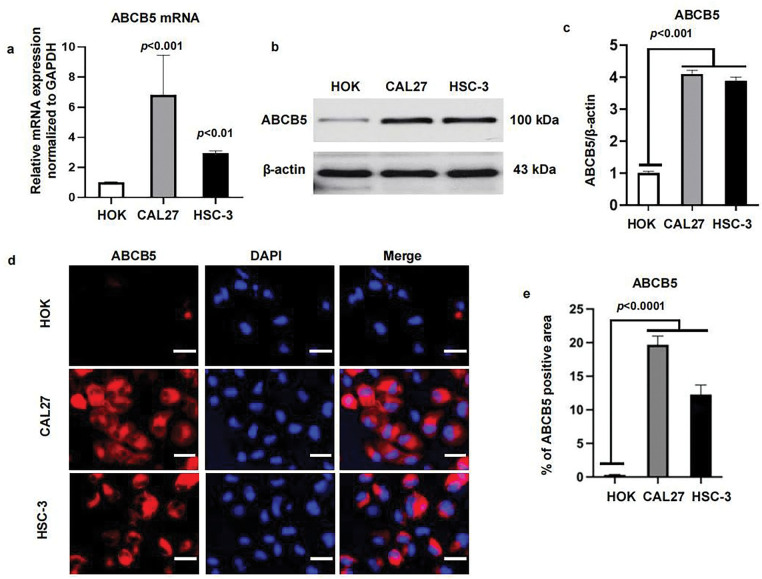
ABCB5 is highly expressed in oral cancer cell lines: (**a–e**) The mRNA and protein expressions of ABCB5 were measured in HOK, CAL27, and HSC-3 using qRT-PCR, western blot, and ICC. (**a**) ABCB5 mRNA level in HOK, CAL27, and HSC-3 was detected by qRT-PCR, *n* = 3 qRT-PCR runs. (**b,c**) The ABCB5 protein level in HOK, CAL27, and HSC-3 was measured by western blot (**b**), the density of ABCB5 was quantified by ImageJ, and β-actin was used as a loading control, *n* = 3 (**c**). (**d**) Representative images of HOK, CAL27, and HSC-3 cells stained with ABCB5 (red), scale bar: 100 μm. (**e**) The ABCB5-positive areas, *n* = 5 fields. Data represent the mean ± SD; one-way ANOVA was used (**a,c,e**)

To study the function of ABCB5 in OSCC, we first determined the levels of ABCB5 in the CAL27 and HSC-3 oral cancer cell lines, and HOK. As shown, the expression of ABCB5 in oral cancer cell lines was significantly upregulated as compared with the HOK, both in gene levels using qRT-PCR (Fig. 1a) and in protein levels identified via western blot (Fig. 1b,c). And a relatively high expression of ABCB5 was detected in CAL27 than that in HSC-3 (Fig. 1a–c). In addition, we visualized the location of ABCB5 in cells via ICC and found that ABCB5 was expressed in the cell cytoplasm and membrane. Moreover, consistent with the western blot findings, high expression of ABCB5 in CAL27 and HSC-3 cells was observed (Fig. 1d,e).

### 3.2 ABCB5 Knockdown Inhibits the Migration and Invasion of 2D OSCC Cells

**Figure 2:**
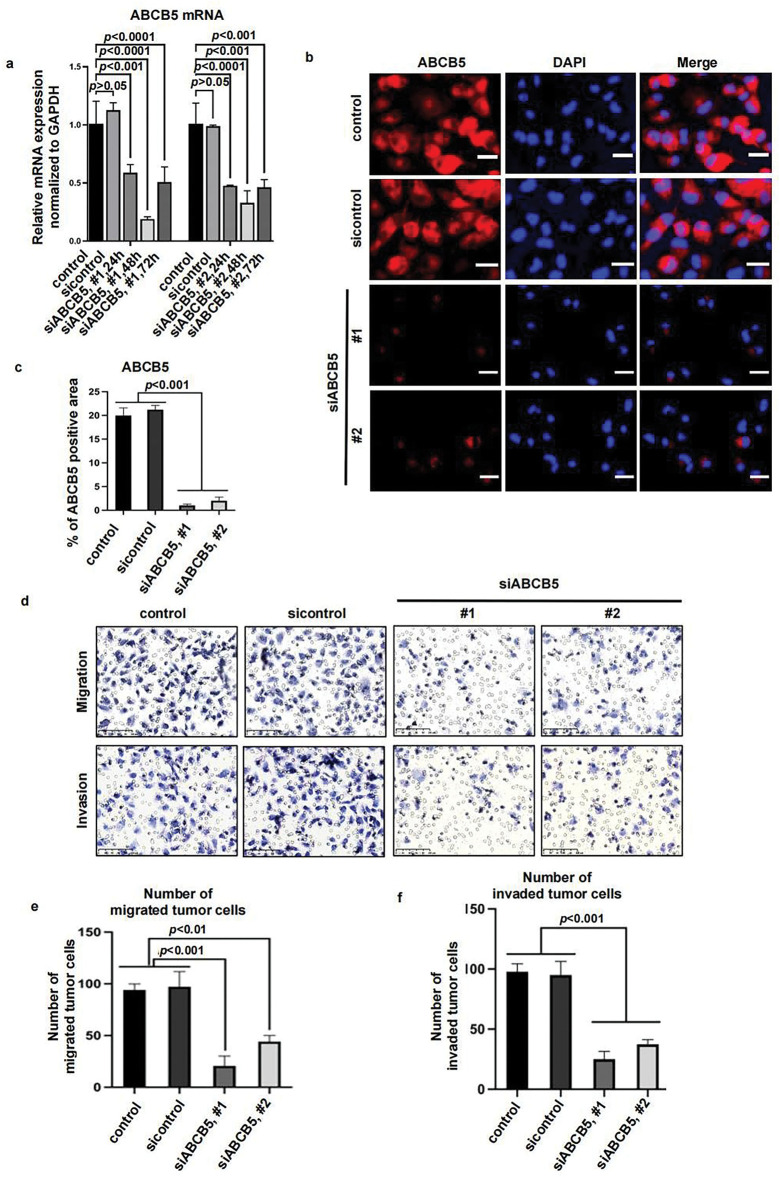
ABCB5 knockdown inhibits the migration and invasion of 2D OSCC cells: (**a–f**) ABCB5 was knocked down in the CAL27 cells using siABCB5, and the migration and invasion ability of the CAL27 cells were evaluated. (**a**) ABCB5 mRNA levels in CAL27 cells transfected with siABCB5 were evaluated using qRT-PCR, *n* = 3 qRT-PCR runs. (**b**) Representative images of CAL27 cells stained with ABCB5 (red), scale bar: 100 μm. (**c**) The ABCB5-positive areas, *n* = 5 fields. (**d–f**) The number of migrated (**d(upper),e**) and invaded (**d(lower),f**) CAL27 cells were imaged, scale bars: 100 μm (**d**) and counted, *n* = 5 fields (**e,f**). Data represent the mean ± SD; one-way ANOVA was used (**a,c,e,f**)

To explore the potential impact of ABCB5 in modulating the motility of OSCC cells, specific ABCB5 siRNAs were used to knock down ABCB5 in the CAL27 cells, which presented a higher ABCB5 expression (Fig. 1a–e). The knockdown efficiency of ABCB5 was confirmed via qRT-PCR (Fig. 2a) at different time points, and the highest knockdown efficiency was detected at 48 h. The protein expression of ABCB5 was examined via ICC at 48 h after siABCB5 transfection (Fig. 2b,c). Research has indicated that ABCB5 was associated with tumor cell migration and invasion [29,30]. Therefore, transwell migration and transwell Matrigel invasion assays were performed to study whether knocking down ABCB5 affects cell migratory and invasive capacities. The results documented that ABCB5 knockdown statistically reduced the number of migrated (Fig. 2d(upper),e) and invaded cells (Fig. 2d(lower),f).

### 3.3 ABCB5 Knockdown Inhibits the Migration and Invasion of 3D Spheroid OSCC

**Figure 3:**
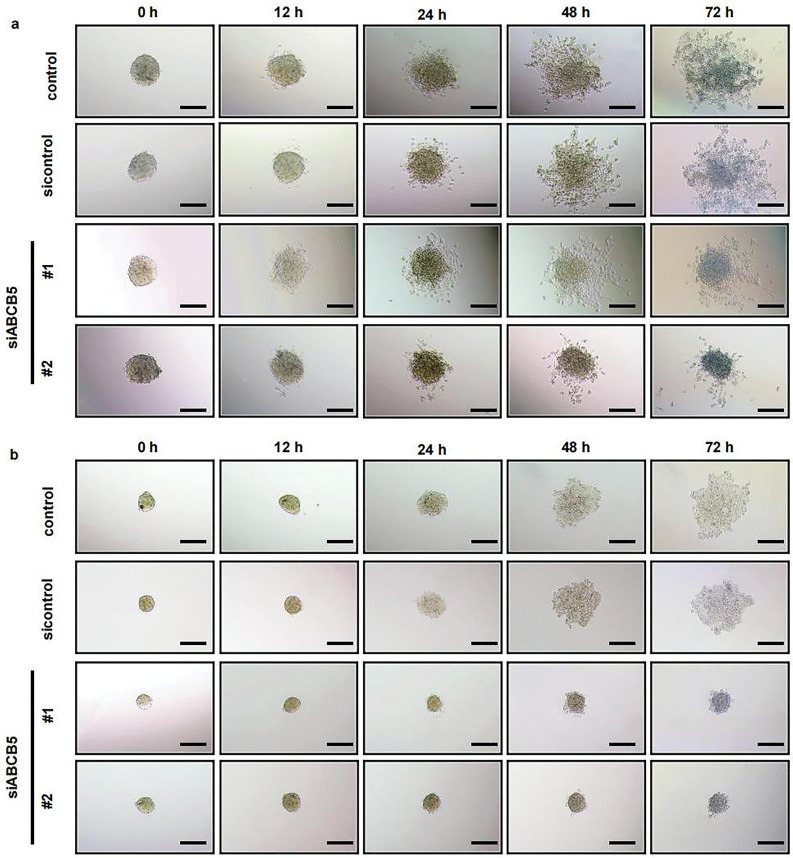

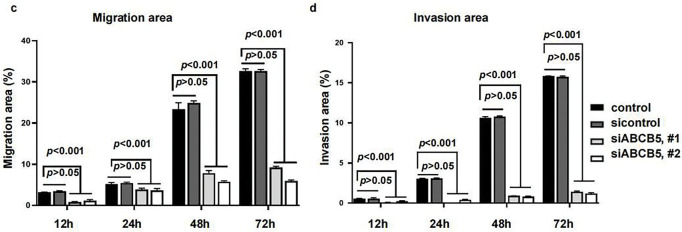
ABCB5 knockdown inhibits the migration and invasion of 3D OSCC spheroid: (**a–d**) The migration and invasion ability of 3D OSCC spheroid were evaluated in the CAL27 cells after transfected with siABCB5. (**a,b**) Representative images of migrated (**a**) and invaded (**b**) 3D OSCC spheroid, scale bars: 100 μm (**a,b**), and the area of cells migrated or invaded were calculated, *n* = 5 fields (**c,d**). Data represent the mean ± SD; one-way ANOVA was used (**c,d**)

Considering the recognized ability of 3D spheroid to recapitulate *in vivo* tumor biology more effectively than the 2D monolayer culture system. 3D spheroid was prepared to evaluate the migration and invasion assays of OSCC cells. ABCB5 silencing significantly reduced the migration (Fig. 3a,c) and invasion (Fig. 3b,d) of OSCC cells in a time-dependent manner, which was consistent with the 2D transwell migration and invasion results. Together, these results suggested that knocking down ABCB5 could inhibit the ability of OSCC cells to migrate and invade, suggesting the prospective involvement of ABCB5 in inducing the migration and invasion abilities of OSCC.

### 3.4 ABCB5 Knockdown Suppresses TGF-**β**-Induced EMT Progression

**Figure 4:**
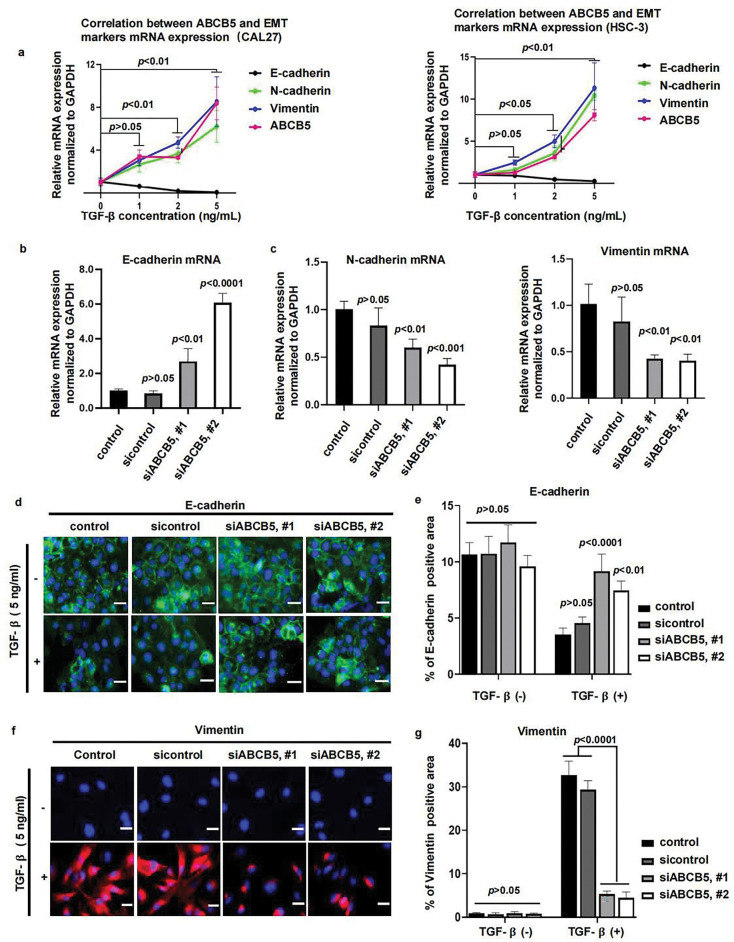
ABCB5 Knockdown suppresses TGF-β-induced EMT: (**a**) The correlations between ABCB5 and epithelial marker E-cadherin and mesenchymal markers (N-cadherin and Vimentin) were observed by qRT-PCR in CAL27 (left) and HSC-3 (right) cells, which were treated with TGF-β in different concentrations, *n* = 3 qRT-PCR runs. (**b,c**) The mRNA expressions of epithelial marker E-cadherin (**b**) and mesenchymal markers (N-cadherin and Vimentin) (**c**) in CAL27 cells were analyzed using qRT-PCR, *n* = 3 qRT-PCR runs. (**d,e**) Representative images of CAL27 cells with or without TGF-β treatment were stained with E-cadherin, scale bar: 100 μm (**d**), and the positive areas of E-cadherin were calculated, *n* = 5 fields (**e**). (**f,g**) Representative images of CAL27 cells with or without TGF-β treatment were stained with Vimentin, scale bar: 100 μm (**f**), and the Vimentin-positive areas were quantified, *n* = 5 fields (**g**). Data represent the mean ± SD; one-way ANOVA was used (**b,c,e,g**); two-way ANOVA was used (**a**).

To investigate whether ABCB5 is correlated with the EMT progression of OSCC, CAL27 and HSC-3 cells were treated with TGF-β, followed by the measurement of ABCB5 and EMT markers. Our results demonstrated a positive association between ABCB5 and mesenchymal markers (N-cadherin and Vimentin), while a negative association between the expression of ABCB5 and epithelial marker E-cadherin in CAL27 (Fig. 4a(left)) and HSC-3 (Fig. 4a(right)) cells was observed. These findings indicate that ABCB5 is positively involved in TGF-β-induced EMT. Moreover, TGF-β initiates EMT progression in CAL27 and HSC-3 cells in a dose-dependent manner, and 5 ng/mL TGF-β resulted in the most obvious difference (Fig. 4a). Furthermore, we found that, compared to HSC-3 cells, the correlation between ABCB5 and EMT markers was more evident in CAL27 cells (Fig. 4a). To further investigate the connection between ABCB5 and EMT progression in OSCC cells, CAL27 cells were transfected with siABCB5, followed by treatment with 5 ng/mL TGF-β. CAL27 cells were transfected with siABCB5 prior to 5 ng/mL TGF-β exposure. ABCB5 knockdown increased the mRNA expression of E-cadherin (Fig. 4b) and decreased the mRNA expressions of N-cadherin and Vimentin (Fig. 4c). In addition, ABCB5 knockdown significantly upregulated the protein expression of E-cadherin (Fig. 4d,e) and decreased the protein level of Vimentin (Fig. 4f,g) in CAL27 cells treated with TGF-β. No obvious difference between the control and ABCB5 knockdown CAL27 cells was evaluated when they were treated without TGF-β. Taken together, these results showed a positive association between ABCB5 and TGF-β-induced EMT.

### 3.5 ABCB5 Was Highly Expressed in the 4NQO-Induced OSCC Model In Vivo

**Figure 5:**
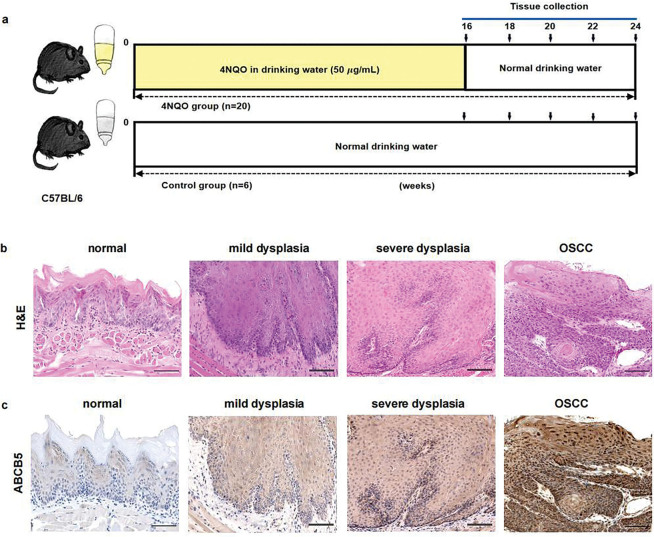

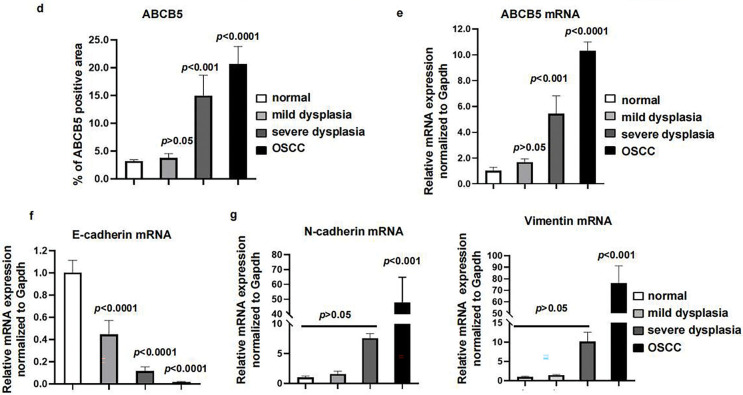
ABCB5 was highly expressed in the 4NQO-induced OSCC model *in vivo:* (**a–g**) The 4NQO carcinogen mouse model was established, and the expressions of ABCB5 and EMT markers were detected. (**a**) Schematic explains the experimental design of the 4NQO carcinogen mouse model. (**b**) Representative images of H&E staining, scale bar: 100 μm. (**c**) Representative image of tongue tissues stained with ABCB5, scale bar: 100 μm. (**d**) Quantification of ABCB5-positive areas, *n* = 5 fields. (**e**) mRNA expression of ABCB5 detected by qRT-PCR, *n* = 3 qRT-PCR runs. (**f,g**) mRNA levels of epithelial marker E-cadherin (**f**) and mesenchymal markers (N-cadherin and Vimentin) (**g**) were evaluated by qRT-PCR, *n* = 3 qRT-PCR runs, *n* = 5–8 mice per group. Data represent the mean ± SD; one-way ANOVA was used (**d,e,f,g**)

To highlight the importance of ABCB5 on the progression of EMT in OSCC. The 4NQO carcinogen mouse model was established as previously reported [41]. Briefly, drinking water containing 50 μg/mL 4NQO was given to the mice to mimic stepwise progression in OSCC patients, and normal drinking water was used as a control (Fig. 5a). Based on H&E staining histopathological assessments, the mice were divided into four groups: the normal group (6 mice), mild dysplasia group (5 mice), severe dysplasia group (7 mice), and OSCC group (8 mice). Normal group specimens displayed intact epithelial architecture with uniform layering and well-defined cellular organization. However, In the mild dysplasia group, the epithelial layer exhibited obvious epithelial thickening and elongated and widened epithelial spikes. In the severe dysplasia group, the epithelium was significantly hyperplastic and thickened, the nucleus was atypical, and the epithelial spikes were fused. In the OSCC group, the cells broke through the basement membrane to reach the lamina propria, the basal cells lost their polarity with enlarged nuclei, and keratin pearls were evident (Fig. 5b). The correlation between ABCB5 and the carcinogenesis process of OSCC was subsequently studied by measuring the mRNA and protein expressions of ABCB5 in the tongue tissues. With the progression of OSCC, ABCB5 expression was significantly increased at the protein (Fig. 5c,d) and mRNA levels (Fig. 5e). In addition, E-cadherin was significantly decreased (Fig. 5f), and N-cadherin and Vimentin were statistically increased (Fig. 5g) with advances in OSCC. Indicating that ABCB5 was positively involved in the EMT progression and contributed to the invasiveness of OSCC.

## 4 Discussion

OSCC is an aggressive epithelial malignant tumor that threatens a global health risk [42]. The high mortality and low survival rates of OSCC were associated with delayed clinical diagnosis and enhanced invasion and metastasis [5,6,14]. To formulate new treatment targets, it is of great significance to understand the occurrence and mechanism of OSCC at a molecular level. In this research, we demonstrated that ABCB5 significantly elevated the migration and invasion abilities of OSCC cells by regulating EMT. Our research indicated that ABCB5 might be a new biomarker for OSCC. Targeting ABCB5 might inhibit the invasiveness and EMT of OSCC.

Previous investigations have shown that ABCB5 level was statistically elevated in several cancers including sinonasal mucosal melanoma [43] and glioblastoma [27]. Consistent with the results of the above studies, our research showed that ABCB5 was overexpressed in OSCC cells as compared with HOK. In addition, we identified the upregulation of ABCB5 in the 4NQO carcinogen mouse model with the dynamic advances in OSCC. Our observations suggested that ABCB5 may be involved in promoting the OSCC progression. Previous studies have demonstrated that ABCB5 overexpression in CSCs associated with tumor progression, chemoresistance and disease relapse in malignant melanoma patients [44]. In addition, another study revealed that ABCB5 promotes tumor vascular invasion and metastasis by activating IL-8/AXL signaling [20]. Collectively, in the current study, we observed a notable reduction in the migration and invasion of 2D OSCC cells and 3D OSCC spheroid after ABCB5 knockdown. Suggesting that ABCB5 was involved in inducing the migration and invasion of OSCC.

ABCB5 promotes angiogenesis, EMT, and tumor metastasis by activating the Hedgehog, Notch, and Wnt pathways [45]. In breast cancer, overexpression of ABCB5 significantly promotes the migration, invasion, and EMT cascade [30]. TGF-β1 reduces the level of E-cadherin and enhances the expressions of N-cadherin and Vimentin to activate EMT via the Smad2/3-dependent pathway [46]. Furthermore, upon EMT, TGF-β treatment could upregulate the ABCB5 expression in breast cancer cells [47]. In addition, TGF-β induced EMT could further increase the migratory and invasive properties of cancer cells [48]. Our investigations observed a reduction in E-cadherin, accompanied by significantly elevated expressions of N-cadherin and Vimentin in OSCC cells. However, the level of EMT markers demonstrated to be the opposite after ABCB5 was knocked down in OSCC cells. Additionally, we obtained consistent data on the positive association between ABCB5 and EMT in the 4NQO carcinogenesis model *in vivo*. These findings suggested that ABCB5 was involved in activating EMT progression, thereby, promoting the migration and invasion of OSCC cells.

The continuous evolution of treatment technology has led to improvements in the therapeutic efficacy of OSCC. However, the prognosis is still not satisfactory, partly due to the chemoresistance [32]. ABCB5 is a drug efflux transporter, which participates in cellular drug efflux thereby enhancing the chemoresistance of tumors. It has been indicated that overexpression of ABCB5 was closely related to poor prognosis in various cancers [49,50]. Higher ABCB5 level increase the chemoresistance to cisplatin in malignant melanoma, suggesting that ABCB5 may be a new target to enhance the drug efficacy of cisplatin [44]. ABCB5 modulates G2/M checkpoint regulators to promote the chemoresistance of glioblastoma [27]. A study found that overexpression of ABCB5 was related to chemoresistance of OSCC [51]. EMT is recognized to increase the chemoresistance of tumor cells [52]. In lung cancer cells, EMT impairs drug sensitivity and promotes acquired resistance to epidermal growth factor-tyrosine kinase inhibitors (EGFR-TKI) [53]. In pancreatic cancer, inhibition of EMT significantly enhanced the chemodrug sensitivity [54]. Metastasis-associated lung adenocarcinoma transcript 1 (MALAT1) enhanced the cisplatin resistance of OSCC via promoting the EMT process [32]. Consistent with previous studies, our data revealed that ABCB5 knocking down significantly induces E-cadherin upregulation concurrent with N-cadherin downregulation in OSCC. These findings indicated that ABCB5 participated in regulating EMT and may additionally facilitate chemoresistance acquisition in OSCC. However, further study is needed to understand the correlations between ABCB5 and chemoresistance in OSCC, as well as the connections between ABCB5 and ABCB5-induced EMT in the development of chemoresistance in OSCC.

Elevated expression of ABCB5 was associated with tumor growth, aggressiveness, multidrug resistance, cancer stemness, and poor overall survival in cancers [18,27,49,55]. Tumor microenvironment (TME) referring to the environment that supports tumor growth and metastasis, is made up of fibroblasts, endothelial cells, cancer cells, immune cells, and extracellular matrix (ECM). TME plays a crucial function in the development and treatment of cancers [56]. Mechanistic investigations revealed that ABCB5 promotes the ability of tumor cells to infiltrate into the blood vessels, which may be regulated by IL-8/AXL signaling [44]. A recent study showed that ABCB5-positive mesenchymal stem cells interact with macrophages and regulatory T lymphocytes to suppress the release of reactive oxygen species and neutrophil extracellular traps [57]. In addition, ABCB5 increased endothelial cell motility and proliferative capacity [58]. Previous research indicated that ABCB5 was involved in inducing the cancer stemness of OSCC [21]. Together, these studies suggested that ABCB5 may be involved in inducing the cancer stemness and the formation of TME, further study is required to study the regulatory mechanism. In addition, this study didn’t incorporate the clinical data, further investigations with clinical samples and analysis are necessary to validate our findings to establish a direct clinical significance of ABCB5 in OSCC progression. While our *in vivo* study demonstrated a correlation between ABCB5 expression and tumor progression, future studies using ABCB5 knockdown or knockout approaches in *in vivo* models to provide mechanistic insights into its role in tumor progression should be addressed. Furthermore, given the diverse subtypes and molecular variations in OSCC, two OSCC cell lines used in the current study may not fully represent the heterogeneity of OSCC, future studies should be performed by including additional cell lines to enhance the generalizability of our findings.

## 5 Conclusion

In conclusion, our findings established that ABCB5 facilitates OSCC progression by enhancing migratory capabilities, invasiveness, and EMT processes. Suggesting that ABCB5 might be a new biomarker for OSCC. Moreover, Targeting ABCB5 might achieve a more favorable therapeutic efficacy for the clinical treatment of OSCC.

## Supplementary Materials



## Data Availability

The datasets supporting the findings of this study can be obtained by contacting the corresponding authors upon reasonable request.

## Supplementary Materials

The supplementary material is available online at https://www.techscience.com/doi/10.32604/or.2025.064276/s1.
