# Supplementary material for: Identifying ATP-Binding Cassette Member B5 as a New Biomarker for Oral Squamous Cell Carcinoma
Source: Oncol Res. 2025 Jul 18;33(8):2037–53. doi: 10.32604/or.2025.064276 (PMC12308245; doi:10.32604/or.2025.064276)
Supplement: Supplementary file 1 [file OncolRes-33-64276-s001.pdf]

Table S1

Primers used in real-time PCR.

Gene	Full name	Primer sequence (F: forward)	Primer sequence (R: reverse)
Homo, ABCB5	Adenosine triphosphate binding cassette subfamily B member 5	5’ACAGCCAAAACTGAGAAAGGAAGC3’	5’GCTCCTCGGGCTATTGCGAA3’
Homo, Vimentin	Vimentin	5’GCCAACTACATCGACAAGGTGC3’	5’TCTCCTCCTGCAATTTCTCCCG3’
Homo, E-cadherin	E-cadherin	5’TAGAGGCTTCTGGTGAAATCGC3’	5’ACCTGACCCTTGTACGTGGTG3’
Homo, N-cadherin	N-cadherin	5’TCAGGCGTCTGTAGAGGCTT3’	5’AAAATCTGCAGGCTCACTGCTC3’
Homo, GAPDH	Glyceraldehyde-3-phosphate dehydrogenase	5’GCCGTCAACGACCCCTTCATTGA3’	5’GGGTGGAGTCGTACTTGAGCATGT3’
Mus, ABCB5	Adenosine triphosphate binding cassette subfamily B member 5	5’GAGGAGAAAAGCCACACACG3’	5’ACTTGTTCTTGCAGAGGTCCAT3’
Mus, Vimentin	Vimentin	5’GTCCGCACATTCGAGCAAAG3’	5’TAGTTGGCGAAGCGGTCATT3’
Mus, E-cadherin	E-cadherin	5’AACCCAAGCACGTATCAGGG3’	5’GAGTGTTGGGGGCATCATCA3’
Mus, N-cadherin	N-cadherin	5’CAGCCGGAGAACAGTCTCCAA3’	5’GAAGCCTCCACAGACGCCTGAA3’
Mus, Gapdh	Glyceraldehyde-3-phosphate dehydrogenase	5’TGTGTCCGTCGTGGATCTGA3’	5’TTGCTGTTGAAGTCGCAGGAG3’
